# A new marine interstitial *Psammogammarus * (Crustacea, Amphipoda, Melitidae) from Gura Ici Island,  off western Halmahera (North Moluccas, Indonesia),  and an overview of the genus

**DOI:** 10.3897/zookeys.128.1661

**Published:** 2011-09-09

**Authors:** Ronald Vonk, Bert W. Hoeksema, Damià Jaume

**Affiliations:** 1 Netherlands Centre for Biodiversity Naturalis (section ZMA), Mauritskade 61, 1092 AD Amsterdam, The Netherlands; 2 Netherlands Centre for Biodiversity Naturalis, P.O. Box 9517, 2300 RA Leiden, The Netherlands; 3 IMEDEA (CSIC-UIB), Instituto Mediterráneo de Estudios Avanzados, C/ Miquel Marquès 21, 07190 Esporles, Balearic Islands, Spain

**Keywords:** Shallow marine interstitial, sandy beaches, genus review, *Psammogammarus*, sexual dimorphism

## Abstract

*Psammogammarus wallacei* **sp. n.** is described from the shallow marine interstitial of a sand and coral rubble beach on the Gura Ici islands (North Moluccas; Indonesia). This is the first record of this circum-tropical genus from SE Asia, with the geographically closest relative inhabiting the Ryukyu archipelago in Japan. The new species is highly distinctive by the display of sexual dimorphism on pleopod II, with the medial margin of the male proximal article of exopod provided with a comb of short, blunt curved spinules; no other representative of the genus is known to display sexually-dimorphic appendages aside of the gnathopods. The new species is also noteworthy by the outline of the palm margin of male gnathopod II, hardly excavated, and by showing a carpus broader than long. An overview of the genus *Psammogammarus* with 14 species to date is provided.

## Introduction

**Figure 1.**
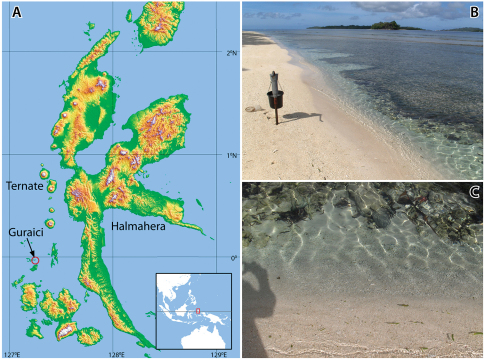
Location and habitat of *Psammogammarus wallacei* sp. n. **A** Gura Ici islands (Guraici = local spelling), low islands consisting of calcareous coral rises and mangrove fringed sand flats **B** groundwater pump with perforated pipe at 50 cm in the sediment (coarse sand and degraded coral) **C** shoreline at low tide, leaving a permanent narrow channel between the beach and the reef flat.

In October and November 2009, a marine expedition was organized by NCB Naturalis, the Research Centre for Oceanography of the Indonesian Institute of Sciences (RCOLIPI), and students of Universitas Khairun, Ternate, North Moluccas. The main goal of the expedition was to investigate the community structure and species composition of coral reef biotas off western Halmahera, in particular around the volcanic islands of Ternate and Tidore. In the wake of this survey a shore party sampled beaches and inland brackish wells for subterranean invertebrates. Several types of coastal sediments were probed, such as beaches of homogeneous black volcanic sand, coral rubble bars, fine silt between stones in bays, and very shallow coral reef flats filled with sand and thick packets of coral fragments. Although the research was based at LIPI’s field station at Ternate, a two day survey was held at the more southwardly located Gura Ici islands (Fig. 1).

Between the copepod and peracarid crustaceans encountered in the thick layers of coarse beach sand in this island group a typical shallow marine interstitial gammaridean amphipod was found, belonging to the melitid genus *Psammogammarus* S. Karaman, 1955. This group is widespread in tropical and subtropical marine beach environments and can only be dug out or pumped from groundwater. Its occurrence begins, from a landward view, in anchialine cave waters in coastal localities, such as known from Bonaire (Vonk and Stock 1987) and the Red Sea (Stock and Nijssen 1965; Jaume and Garcia 1992), down to depths of around 1200 m at the Balearic Sea slope (Cartes and Sorbe 1999).

It is remarkable that *Psammogammarus* has not been found in Australia despite quite intensive sampling there in exactly the same shallow marine sediments in which it occurs in many other tropical seas of the world (Yerman and Krapp Schickel 2008).

## Material and methods

The marine stygofaunal research carried out in the North Moluccas concentrated on collecting subterranean species in diverse habitats at different places on the islands. We sampled in coastal areas, wells, small brackish lakes, beaches, and mangrove fringes of Ternate, Hiri, Tidore, Maitara, Guraici Islands, and the west and east side of northern Halmahera. Sampling gear consisted of a biophreatical Bou-Rouch groundwater pump and steel pipes (see Bou 1974), a Cvetkov net (Cvetkov 1968), a dip net, and a shovel. On beaches the pump was placed near to the waterline. When the marine groundwater flow was not steady and the pipe holes or pump cylinder were clogged with sand and silt, the pump was placed directly in the sea. Low tide was the preferable time to sample but since the tidal difference was only 1–2 m and locations logistically restricted, sampling was performed at all tides. In some cases, ground water was filtered that accumulated in holes dug with a hand shovel. In wells we used a 0.30 mm mesh size Cvetkov net, and in scooping up sand from shallow marine reef flats hand nets of various mesh sizes. The 2% formalin-preserved samples (short time for hardening tissue) were sorted later in the LIPI Ternate field station laboratory under a dissecting microscope and transferred to 70% ethanol. Some samples with abundant specimens were directly stored in 96% ethanol. Before study, specimens were treated with lactic acid to soften the cuticle and remove internal tissues to facilitate observation. Drawings were prepared using a camera lucida on Olympus BH2 and Leica DM 2500 microscopes equipped with Nomarski differential interference contrast. Material preserved on slides was mounted in lactophenol and the coverslips sealed with nail varnish. Body measurements were derived from the sum of the maximum dorsal dimensions (including telescoped portions) of head, pereonites, pleosomites and urosomites, and exclude telson length. Material is deposited in the Crustacea collection of the Zoological Museum of the University of Amsterdam (ZMA).

Following Watling (1989), the term “spine” in descriptions is restricted for rigid armature elements with a hollow central core that do not articulate basally to the body integument. Gnathopods I and II, and pereiopods V to VII appear abbreviated elsewhere as G1–G2 and P5–P7, respectively; uropods I–III, as U1–U3; exp1 and exp2 denote proximal and distal segment, respectively, of the 2-segmented exopod of uropod III.

## Taxonomy

### Order Amphipoda Latreille, 1816. Suborder Gammaridea Latreille, 1802. Family Melitidae Bousfield, 1973. Genus Psammogammarus S. Karaman, 1955

#### Psammogammarus wallacei sp. n.

urn:lsid:zoobank.org:act:5E679036-2477-4485-BAD0-6F6D452D2288

http://species-id.net/wiki/Psammogammarus_wallacei

**Figure 2.**
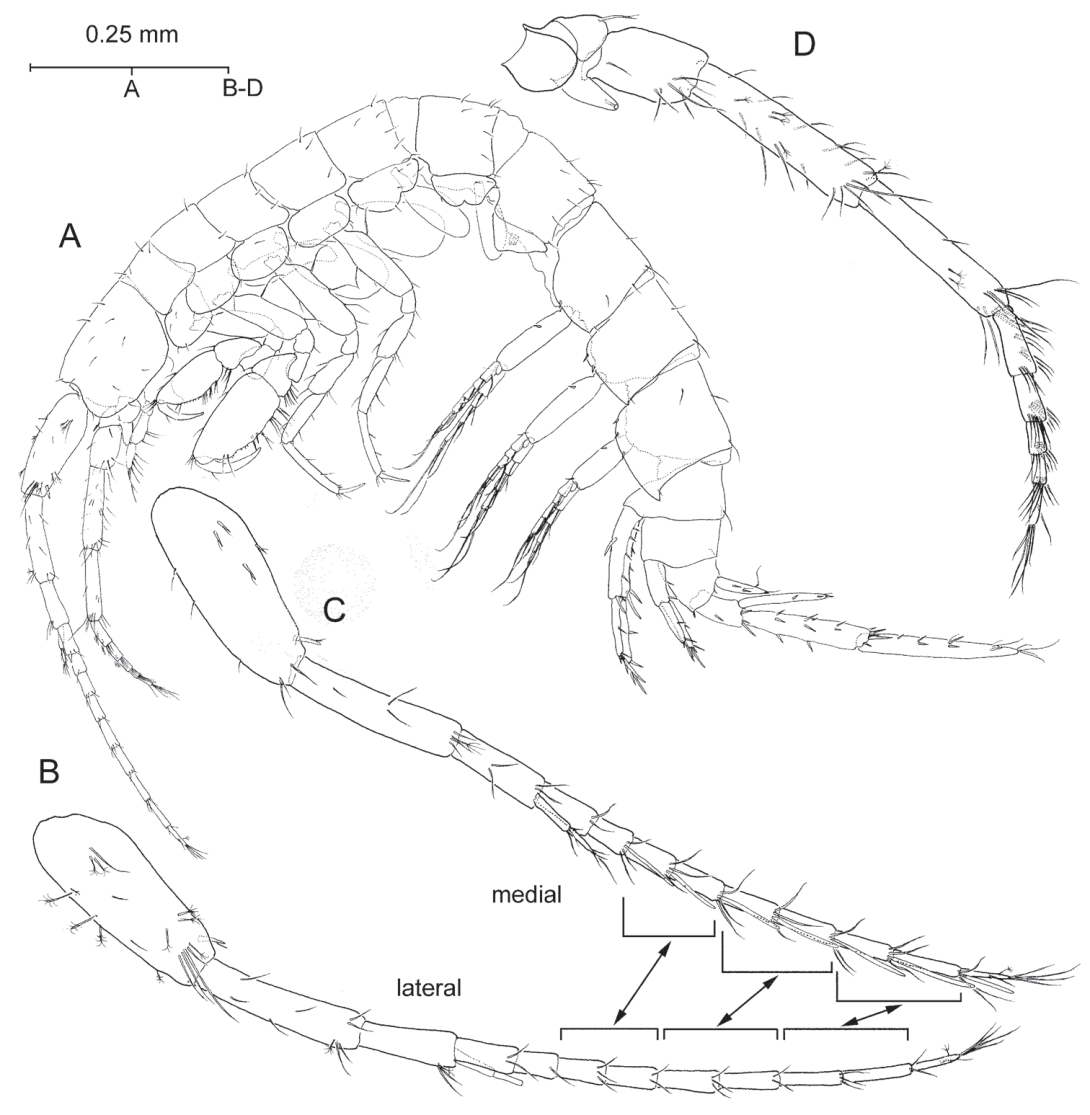
*Psammogammarus wallacei* sp. n., male paratype 2.53 mm, P5-P7 wanting. **A** body, lateral **B** left antennule, lateral **C** same showing medial armature **D** right antenna, lateral. Arrows pointing at serially-homologous pairs of articles of main flagellum of antennule.

Figs 2 [Fig F3] [Fig F4] [Fig F5] [Fig F6] [Fig F7] 8 

##### Material examined.

Collected by R. Vonk and Mr. Sumadijo, 9 November 2009.Gura Ici islands, north beach of Pulau Lelei, thick coral rubble bar at waterline bordering shallow reef flat (stn. 09–60; 0°01'38.64"N, 127°14'38.53"E); B-Rh pump placed on slope of coral bar, pipe 50 cm depth, 50 l filtered. **Holotype:** Adult male (with penile papillae) 2.65 mm retaining all limbs except U3, completely dissected and mounted on single slide [ZMA, amph. 206076]. **Paratypes:** five adult males of 2.53, 2.54, 2.55, 2.57 and 2.63 mm; two brooding females (oostegites developed, setose) 2.60 and 2.98 mm; two juveniles 1.79 and 1.87 mm. All in single vial [ZMA, amph. 206075].

##### Diagnosis.

 Male G2 palm margin only slightly excavated, devoid of mid-palmar strong robust setae; carpus broader-than-long. Uropod III exp2 longer than exp1; endopod elongated, more than 50% length of exp1. Protopod of U1 with one basofacial robust seta. Distomedial angle of U2 protopod provided with transverse comb of 4 robust setae. Telson with two lateral and one distal robust setae. Posteroventral angle of epimeral plate III pointed but weakly produced. Armature (robust setae) on ventral margins of epimeral plates as 1-(2 or 3)-3. Coxal endite (= inner plate) of maxillule provided with 6 setae; basal endite (= outer plate) with 9 robust setae. Oblique row on inner plate of maxilla composed of 4 setae. Basal endite (= inner plate) of maxilliped provided with three robust setae; ischial endite (= outer plate) with 4. Basis of P7 weakly expanded, with sub-parallel anterior and posterior margins. Pleopod II sexually-dimorphic.

##### Etymology.

Species name after the 19th century British naturalist Alfred R. Wallace, who was based in Ternate during his explorations of the Moluccas.

##### Description.

 **Adult male.** Eyeless. *Body* (Fig. 2A) elongate and slender, unpigmented, somites devoid of relevant armature or sculpturing except for robust seta present on posteroventral angle of urosomite III (Fig. 8B). *Head* lacking rostrum; lateral lobes evenly rounded; antennal sinus hardly indicated, unnotched. *Pereiopodal coxae* narrow, hardly overlapping. *Epimeral plates* (Fig. 8A) with acute posteroventral angles, that of epimeral plate III more produced than rest; armature of ventral margin of plates (flagellate robust setae) as 1-3-3 or 1-2-3; posterior margin of plates each with single simple seta implanted adjacent to posterodistal angle.

*Antennule* short, about half as long as body length (Fig. 2A). Peduncle segments relative length as 100: 77: 43; proximal segment provided with stout flagellate robust seta subdistally on ventromedial margin (Fig. 2B, C). Main flagellum about as long as peduncle, with armature on medial and lateral margins exactly as depicted; armature of two most proximal articles and terminal article differing from rest as figured. Accessory flagellum 2-articulate, overreaching distal margin of proximal article of main flagellum.

*Antenna* (Fig. 2A, D) shorter, about three-quarters length of antennule. Gland cone slender, straight, pointing anteriorly; relative length of three distal segments of peduncle as 48: 100: 88. Flagellum short, slightly longer than distal segment of peduncle.

*Labrum* trapezoidal. *Paragnaths* (Fig. 8D) with distinct, well developed inner lobes; outer lobes each with 4 stout tricuspidate setae on tip.

**Figure 3. F3:**
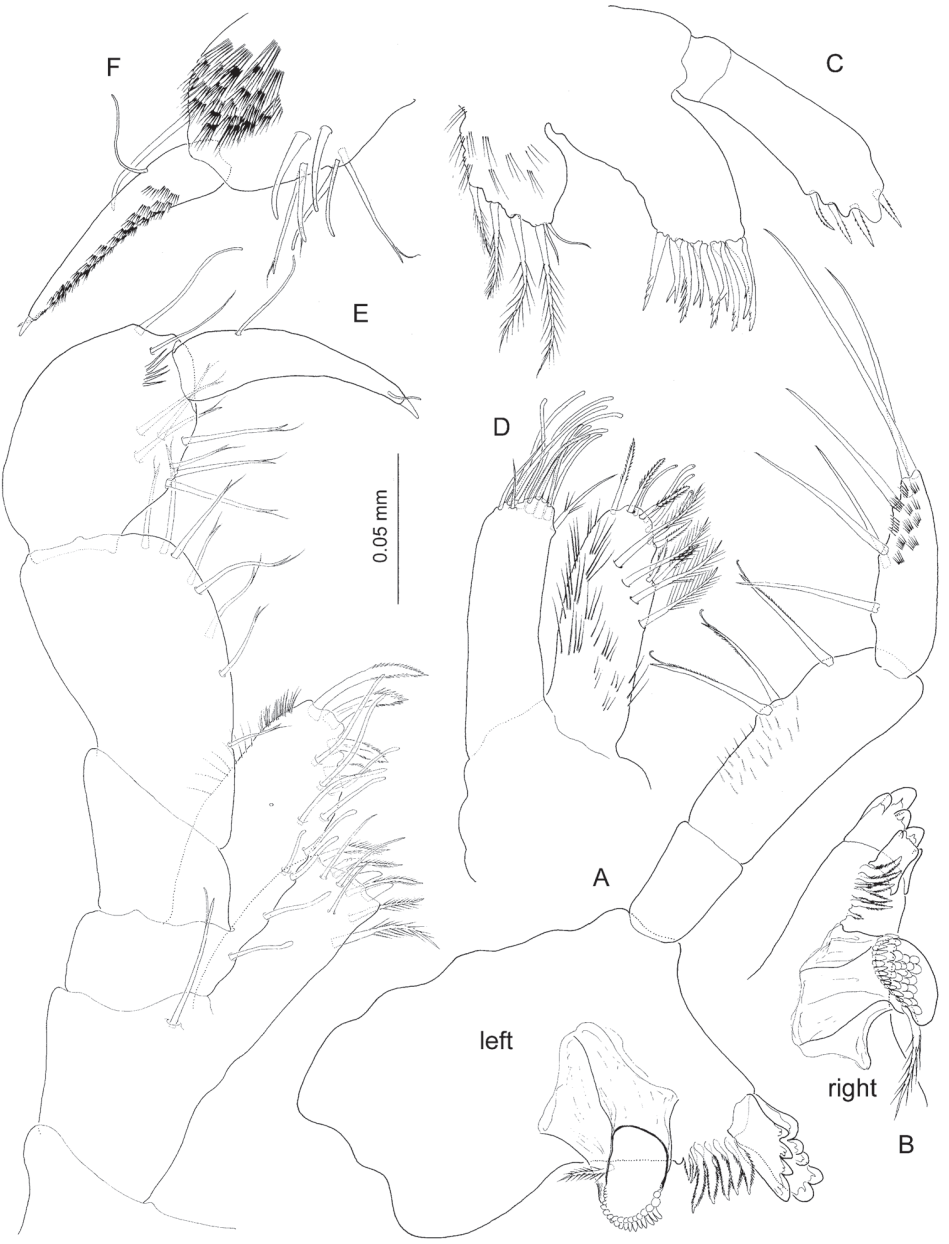
*Psammogammarus wallacei* sp. n., male holotype. **A** left mandible **B** right mandible **C** maxillule **D** maxilla **E** maxilliped, posterior **F** inset of distal segments of palp of latter, anterior.

*Left mandible* (Fig. 3A) incisor with 5 rounded teeth, lacinia with 4 teeth; spine row composed of 7 elements; molar columnar, molar seta shorter than right mandible counterpart. Palp 3-segmented, distal segment shorter than middle segment, provided with 6 stiff simple setae along medial margin but not in a regular row; middle segment with three flagellate stiff setae on medial margin. *Right mandible* (Fig. 3B) differing from left counterpart in almost quadrate lacinia with distal margin finely denticulated except for two larger rounded denticles at one angle; spine row comprising 5+3 elements.

*Maxillules* (Fig. 3C) symmetrical, coxal endite (= inner plate) with 4+2 marginal setae; basal endite (= outer plate) with 9 robust setae distally, 4 of which bicuspid and placed conforming an inner row, rest denticulated and conforming outer row. Endopod (= palp) faintly 2-segmented, distal segment slightly expanded distally, with 4 broad, short denticulated robust setae on distal margin, and 1–2 denticulated setae subdistally on outer surface of segment.

*Maxilla* (Fig. 3D) inner plate with oblique row of 4 plumose setae; distal margin with 4 pinnate setae and 4 simple setae with blunt tip provided with pore; two pinnate setae subdistally on medial margin of plate as figured. Outer plate distal margin with 10 simple setae with blunt tip, two shorter, ordinary simple setae, plus seta with a few pinnules proximally as figured.

*Maxilliped* (Fig. 3E) basal endite (= inner plate) subrectangular, straight distal margin provided with three short subtriangular robust setae, two of which smooth, third with two rounded denticles; other armature on endite comprising subterminal oblique row of 5 pinnate setae on anterior surface, and three simple setae subterminally on posterior surface. Ischial endite (= outer plate) with convex outer margin; straight inner margin with 6 simple setae with blunt tip; distal margin oblique, with 4 pectinate robust setae. Other relevant armature on endite comprising submarginal row of 4 simple setae with blunt tip running subparallel to inner margin on posterior surface. Merus-dactylus (= palp) as in Fig. 3E, F, with claw (= dactylus+unguis) as long as propodus.

**Figure 4. F4:**
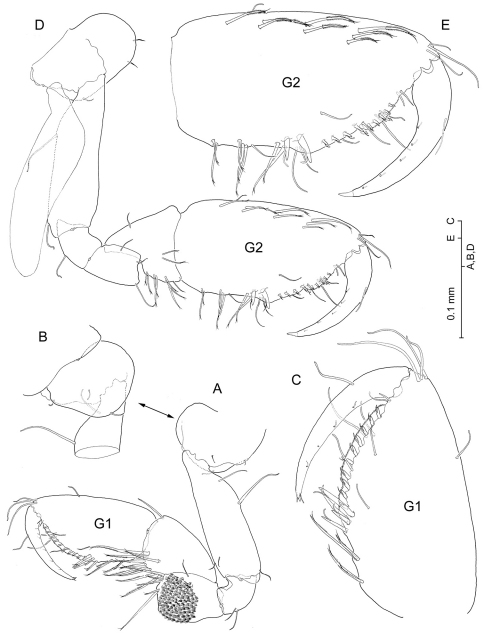
*Psammogammarus wallacei* sp. n., male holotype. **A** right gnathopod I, medial **B** inset of coxa, lateral **C** palm, medial **D** left gnathopod II, medial; palm, medial. Notice that C and E are not at same scale.

**Figure 5. F5:**
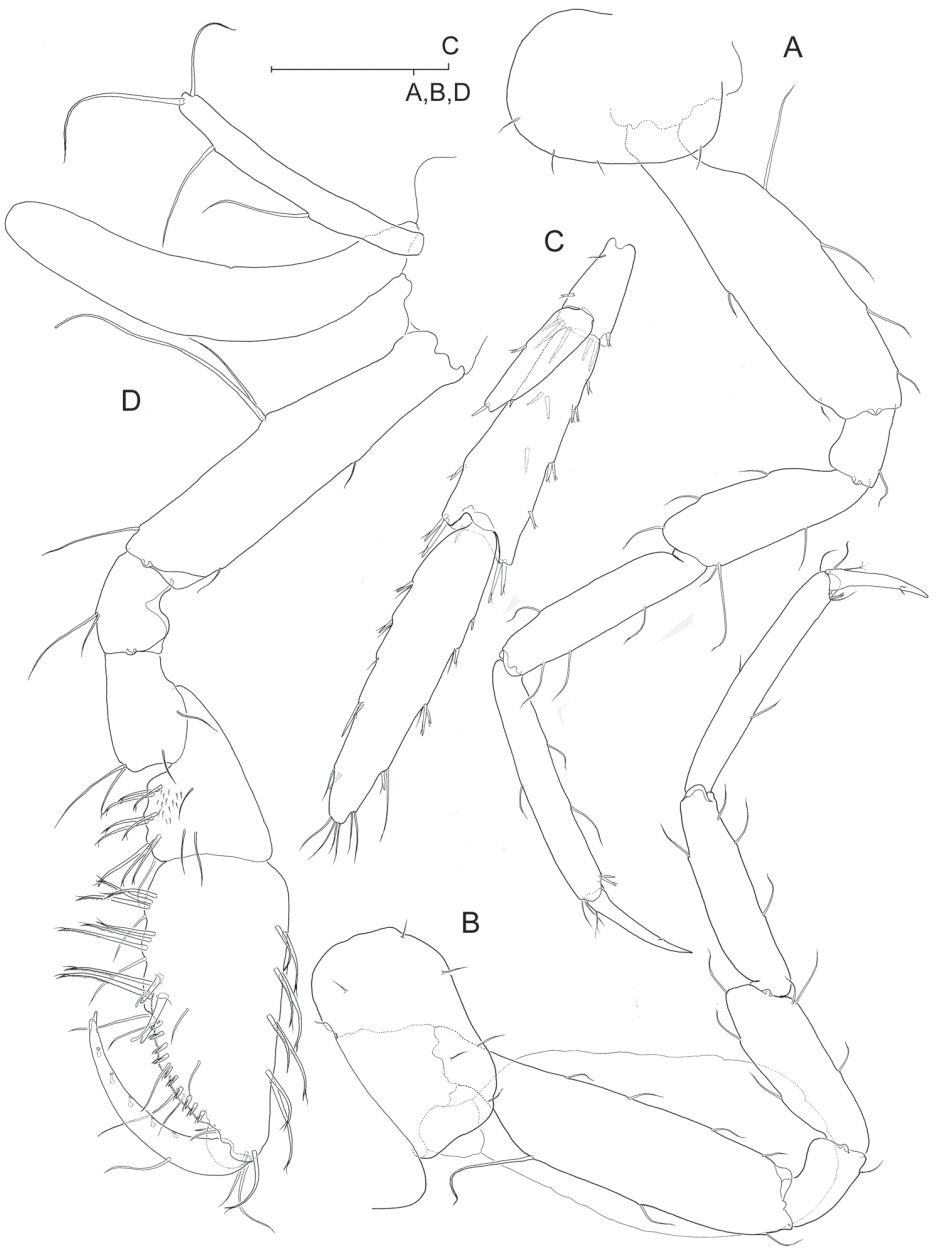
*Psammogammarus wallacei* sp. nov., male holotype. **A** left pereiopod III with coxal gill omitted, lateral **B** right pereiopod IV, lateral **C** right uropod III, dorsal **D** left gnathopod II of female paratype 2.98 mm showing coxal gill and oostegite, coxal plate omitted, medial. [Scale bars: 0.25 mm (C); 0.1 mm (A, B, D)]

**Figure 6. F6:**
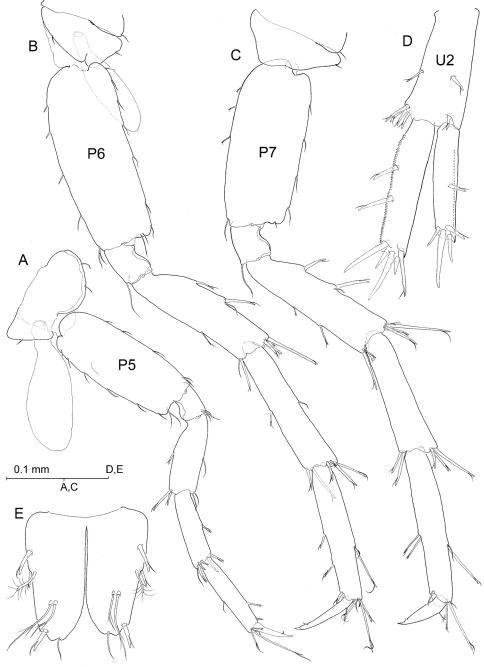
*Psammogammarus wallacei* sp. n., male holotype. **A** right pereiopod V, lateral **B** left pereiopod VI, lateral **C** left pereiopod VII, lateral **D** right uropod II, posterior **E** telson, dorsal.

*Coxal gills* on gnathopod II and pereiopods III-VI (Fig. 2A); gills II (Fig. 4D) and III-IV (Fig. 5B) each longer than basis of corresponding pereiopod, gills V-VI (Fig. 6A, B) shorter than basis; gill VI reduced. Gill II narrow, sausage-shaped, rest ovoid; all provided with short stalk.

*Gnathopod I* (Fig. 4A-C) propodus clearly longer than carpus, elongate (1.9 times as long as broad), with parallel anterior and posterior (= lateral and medial) margins. Palm margin oblique, convex, finely serrated, with submarginal row of about 10 short flagellate robust setae along medial side. Palm angle not produced, ordinarily with two unequal flagellate robust setae, but extraordinarily with 3–4 (Fig. 4C). Coxa (Fig. 4B) slightly broader than long, with evenly rounded anterior margin; posterior margin slightly excavated.

*Gnathopod II* (Fig. 4D) propodus elongate (1.9 times as long as broad) with subparallel anterior and posterior margins; palm angle hardly produced, with two unequal flagellate robust setae; palm margin (Fig. 4E) finely serrated, half adjacent to palm angle hardly excavated, with submarginal row of ca. 9 short flagellate robust setae along medial side. Merus posterodistal angle slightly produced, but lacking pointed tip. Carpus triangular, short, less than half length of propodus.

*Pereiopods III-IV* (Fig. 5A, B) subsimilar, with coxae much broader than long, subrectangular, expanded anteriorly into evenly rounded lobe and posterior margin not excavated, straight.

*Pereiopods V-VII* unequal in length, P5 (Fig. 6A) shortest, P6 (Fig. 6B) longer than P7 (Fig. 6C). Basis of each pereiopod only moderately expanded, with subparallel margins and with posterodistal angle distinct but not overhanging. Nail (dactylus + unguis) of P6 more slender and clearly longer than those of P5 and P7. Unguis reduced in all limbs. Coxa V with expanded, evenly rounded anterior lobe.

**Figure 7. F7:**
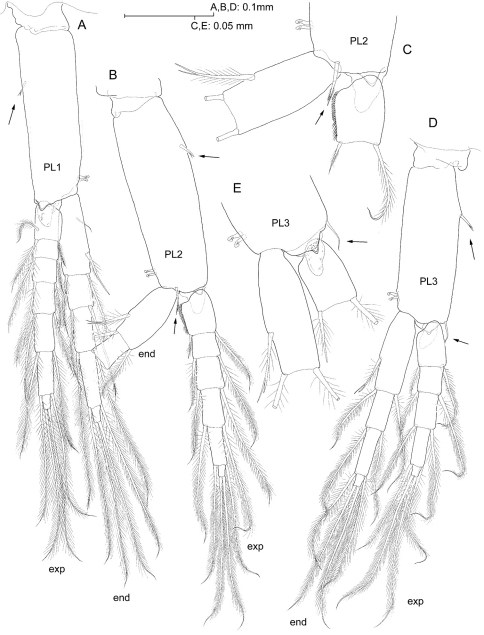
*Psammogammarus wallacei* sp. n., male holotype. **A** left pleopod I, posterior **B** left pleopod II with distal portion of endopod omitted, endopod unnaturally bent to expose medial margin of proximal article of exopod, anterior view **C** detail of latter, anterior **D** left pleopod III, anterior **E** detail of distal portion of protopod of right pleopod III, posterior. Arrows pointing at armature elements of protopod.

*Pleopods* progressively shorter towards posterior, biramous, rami multi-articulate, apparently similar at first sight, but differing remarkably in minute details as follows. Protopods each with two retinacles, and with flagellate robust seta placed proximo-laterally on anterior surface of segment (pleopods I-II; Fig. 7A, B), or on lateral margin (pleopod III; Fig. 7D). Protopod of pleopod I devoid of any other armature; protopod of pleopod II with seta on anterodistal margin (Fig. 7B, C); protopod of pleopod III with seta on distolateral angle (Fig. 7D, E). Short, subtriangular process protruding posterodistally on each protopod, those on pleopods I-II with smooth surface (Fig. 7A, C), that on pleopod III microspinulate (Fig. 7E). Proximal article of endopod of pleopod I with one reduced smooth seta proximally on anterior surface (Fig. 7A); seta absent from rest of pleopods (Fig. 7B, D); proximal seta on medial margin of article apparently unicuspid (Fig. 7A), vs. seta bifid on pleopods II-III (Fig. 7C, E). Proximal article of exopod of pleopod II sexually-dimorphic: proximal two-thirds of medial margin with row of short, curved denticles with rounded tip, and row of ordinary setules along distal third of margin (Fig. 7B, C).

**Figure 8.**
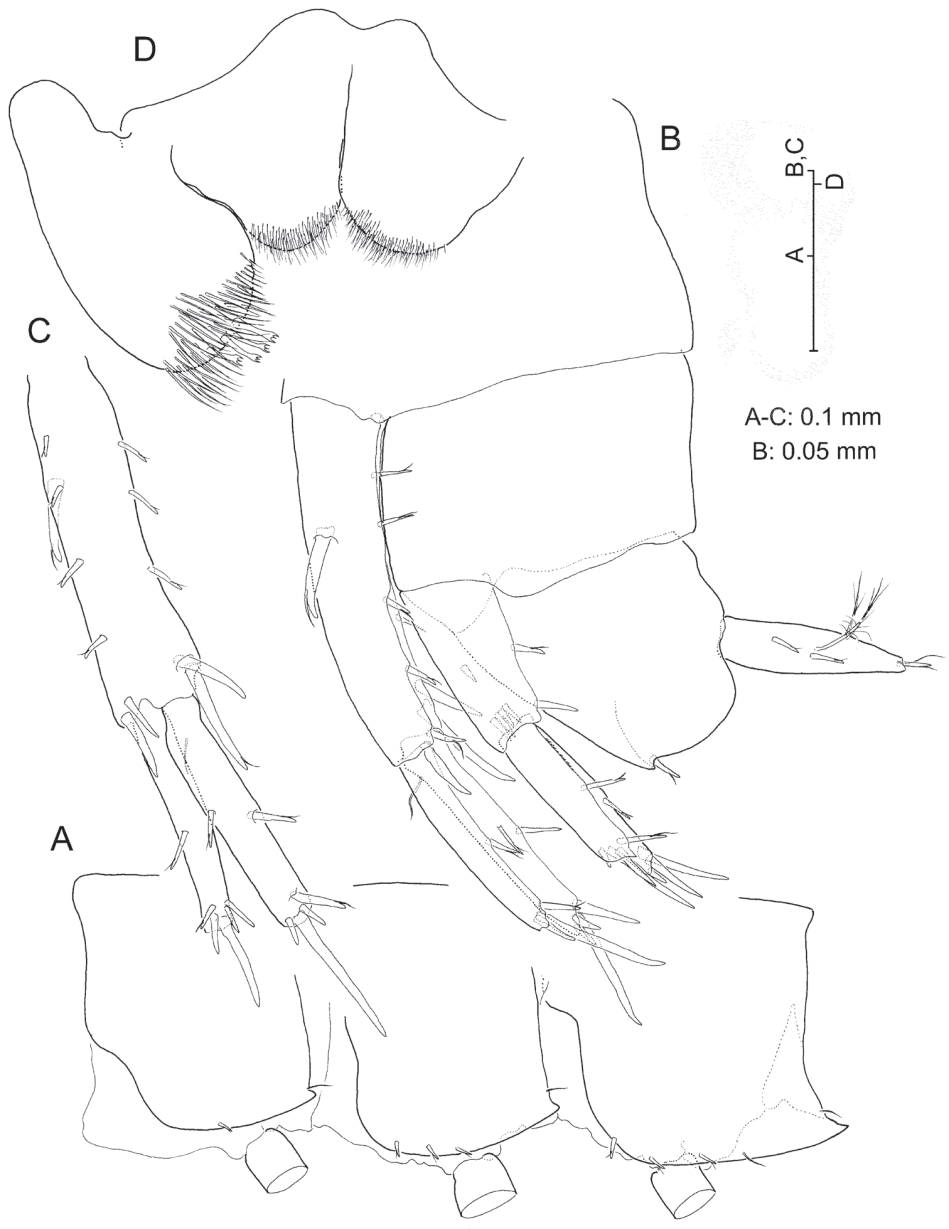
*Psammogammarus wallacei* sp. n., male holotype. **A** left epimeral plates, lateral **B** urosome, lateral (uropod III wanting) **C** left uropod I, posterior **D** paragnaths.

*Uropods I-II* strongly dissimilar in length (Fig. 8B), biramous, exopod shorter than endopod, and with margins of both protopod and rami provided with flagellate robust setae. *Uropod I* (Fig. 8C) protopod much longer than exopod, provided with stout basofacial robust seta and with pair of robust setae on each posterodistal angle, medial pair appreciably longer than lateral counterpart. Posterolateral margin of segment provided with 4 short robust setae, posteromedial margin with three. Exopod with 5 distal robust setae and with robust seta on posterolateral margin; endopod with 4 distal setae and with robust seta about midway on both margins, aside of reduced simple seta on anteroproximal surface of segment. *Uropod II* (Fig. 6D) protopod about as long as exopod, with one robust seta on distolateral angle and with transverse row of 4 robust setae on distomedial angle (see Fig. 8B). Margins of segment each with single robust seta about midway. Exopod with 4 distal robust setae and robust seta about midway of posterolateral margin; endopod with 5 distal robust setae and two robust setae along posteromedial margin. Lateral margin of exopod and medial margin of endopod each minutely serrated (Fig. 6D). *Uropod III* (Figs 2A; 5C) strongly elongated, all segments somewhat flattened, foliaceous, provided with numerous flagellate robust setae as figured. Protopod short; proximal segment of exopod about 2.2 times as long as protopod, distal segment exceedingly longer (1.5×) than proximal segment. Endopod elongate, pointed, much longer than protopod and attaining about 57% length of proximal exopodal segment; one robust seta on tip.

*Telson* (Fig. 6E) cleft almost until base, slightly longer than broad, tip of each lobe shallowly excavated, provided with simple seta. Three flagellate robust setae on each lobe, one placed subdistally while other two proximally on lateral margin as figured. One penicillate seta disposed adjacent to one of lateral robust setae as in Figs 8B and 6E. Pair of long setae on dorsal surface of each lobe as figured.

**Adult female.** As male except for *gnathopod II*, which displays more elongated carpus and evenly convex palm margin of protopod (compare Figs 4D, E and 5D). In addition, proximal article of exopod of *pleopod II* unmodified. *Oostegites* present on gnatopod II and pereiopods III-V, linear, provided with few sparsely set marginal setae (Fig. 5D). Condition of *uropod III* unresolved since none of two females collected retained it.

##### Remarks.

The combined display of a basic gammaridean body plan, linear oostegites, antennae without calceoli, no sternal gills, G1 smaller than G2, both lacking integumentary rugosities, G1 of melitoid type, dispariramous uropod III with a reduced endopod and a 2-segmented exopod with both segments highly elongated and about the same length, and paragnaths with distinct inner lobes, relates the new taxon from Indonesia with a reduced cluster of melitid genera of mostly stygobytic habits known as the Eriopisa-group. The discrimination between several of the components in this complex proved to be controversial (Karaman and Barnard 1979; Stock 1980; Karaman 1984; Stock and Sánchez 1987), holding currently *Eriopisa*, *Flagitopisa*, *Nedsia*, *Norcapensis*, *Psammogammarus*, *Tunisopisa* and *Victoriopisa*.

The new taxon differs markedly from *Victoriopisa* Karaman & Barnard, 1979, a genus from coastal lagoons and marine shallow waters comprising 9 species, in displaying the proximal articles of the antennary flagellum not fused; the ventral margin of epimeral plate II devoid of a row of long setae; the P7 basis not broadly expanded, similar to P6; and the propodus of G1 longer than carpus (vs. carpus equal or longer than propodus in *Victoriopisa*) (see Lowry and Springthorpe 2005 and references therein; Ortiz and Lalana 1989; Lim et al. 2010).

*Eriopisa* Stebbing, 1890, comprising two species, viz. *Eriopisa elongata* (Bruzelius, 1859) from the deep Atlanto-Mediterranean and *Eriopisa incisa* McKinney, Kalke and Holland 1978 from shallow muddy bottoms on the Gulf of Mexico, differs in the notched head lobe; the forwardly pointed coxal plate of G1; the broadly expanded basis of P7, dissimilar to P6; the carpus of G1 as long as or longer than propodus; and the epimeral plate II bearing a row of long setae along ventral margin (McKinney et al. 1978; Lincoln 1979). Two taxa currently included in *Eriopisa*, viz. *Eriopisa inaequicaudata* Ledoyer, 1982 from the Tulear reef in Madagascar and *Eriopisa mochimae* van der Ham & Vonk, 2003 from a sandy beach in Venezuela and coral rubber bars in Curaçao, do not display the diagnostic forwardly pointed coxa I, notched head lobe, setose ventral margin of epimeral plate II, and the P7 basis is similar to P6; they should not be considered as members of the genus, nor even of the *Eriopisa*-group since their U3 exp2 is hardly elongated (Ledoyer 1982; van der Ham and Vonk 2003).

*Flagitopisa* Karaman, 1984, comprising two species from wells and river alluvia in the Philippines, differs in the presence of sternal gills on the first pleonite; the uniarticulate condition of the antennulary accessory flagellum; the high number of robust setae on the basal endite of maxilliped (11 vs. 3 in the new taxon, and up to 6 in the rest of taxa of the *Eriopisa*-group); and the G1 carpus, which is longer than propodus (Sawicki et al. 2005).

*Nedsia* Barnard & Williams, 1995, including 11 species from Australian inland groundwaters, displays a G1 carpus longer than propodus; a broadly expanded, foliaceous U3 exopod; and a 2-segmented mandibular palp (see Bradbury 2002 and references therein).

*Norcapensis* Bradbury & Williams, 1997, a monotypic genus from subterranean waters of W Australia, differs in the fusion of the proximal articles of the antennal flagellum; the carpus of G1 longer than propodus; and the huge G2 propodus, with a short posterior margin and a strongly oblique palm margin.

*Tunisopisa* Stock, 1980, a monotypic genus from wells in Tunisia, displays a non-elongated exp2 of U3 but is traditionally included in the *Eriopisa*-group. This taxon shows a presumed unsegmented maxillulary endopod; a broadly expanded, foliaceous U3 exopod; a G1 propodus longer than carpus; and a peculiar palm of G1, provided with a series of transverse integumentary ridges (Gauthier 1936).

The new Indonesian species is assigned to *Psammogammarus* after its P7 basis, similar to P6, and the proportions of the two distal segments of the mandibular palp, with segment 3 much shorter than segment 2. Only some species in *Nedsia* among members of the *Eriopisa*-group show a slender or weakly broadened basis of P7 approaching the nearly linear basis of P7 of the new taxon, but all *Nedsia* members display a 2-segmented mandibular palp.

*Psammogammarus wallacei* sp. n. differs at first glance from a highly distinctive group of three species of non-interstitial habits, inhabitants of anchialine caves and wells, namely *Psammogammarus burri* Jaume & Garcia, 1992 from the Balearic Is., *Psammogammarus longidactylus* Vonk & Stock, 1987 from Bonaire (Netherlands Antilles), and *Psammogammarus longiramus* (Stock & Nijssen, 1965) from Entedebir Is. (Red Sea). This cluster is characterised by the common display of a male G2 with non-excavated, evenly convex palm margin and an elongated, longer-than-broad carpus.

*Psammogammarus gracilis* (Ruffo & Schiecke, 1975), from the shallow infralittoral interstitial medium of Malta (Mediterranean), shows a highly characteristic bisinusoid conformation of the male G2 palm margin. This feature differs markedly from the condition displayed in the rest of *Psammogammarus*, including the new species, where the palm margin is either excavated or evenly convex.

A third cluster of *Psammogammarus*, of interstitial habits, displays a male G2 palm margin strongly excavated, namely: *Psammogammarus coecus* S. Karaman, 1955 from the western Mediterranean and Adriatic shallow infralittoral; *Psammogammarus garthi* (Barnard, 1952) from a tidal pool at Baja California (Mexico); *Psammogammarus initialis* Stock & Sánchez, 1987 and *Psammogammarus stocki* Vonk, 1990, both from beaches and tidal pools at Tenerife (Canary Is.); *Psammogammarus mawatarii* Tomikawa, Kakui and Yamasaki 2010 from a tidal pool in southern Japan; and *Psammogammarus spinosus* Stock & Vonk, 1992 from a beach at the Cape Verde Islands. Additional remarkable differences between these and other *Psammogammarus* species vs. *Psammogammarus wallacei* sp. n. are shown in Table 1. Intraspecific variation has been accounted for. For instance *Psammogammarus burri* possesses the rather high number of 19 setae on the coxal endite (=outer plate) of the maxillule (character 13, Table 1). However, this number is fixed within the other 6 specimens used for this study. Another research that focussed on variation within bogidiellid amphipods also confirmed only small variation is present in this feature – in one case out of seven 6 spines present instead of 7 (Vonk et al. 1999).

Only *Psammogammarus caesicolus* Stock, 1980 from Curaçao and Bonaire displays a shallowly excavated male G2 palm margin approaching the condition found in the new species. This species shares also with the new taxon the display of a reduced armature on both the coxal endite of the maxillule and the inner plate of the maxilla, a comb of 4 robust setae on the distomedial angle of U2 protopod, an elongate U3 endopod, and a robust seta on tip of telson (see Table 1). But they differ markedly in the exp2 of U3, which is shorter than exp1 in *Psammogammarus caesicolus* vs. longer in the new species; in the presence of 2–3 basofacial spines on the protopod of U1 in *Psammogammarus caesicolus* vs. only one in the new species; in the armature of epimeral plates, devoid of robust setae in *Psammogammarus caesicolus* vs. a 1-(2 or 3)-3 arrangement in the new species; and in the armature of palm angle of G2 in both sexes, with 4 robust setae in male and three in female *Psammogammarus caesicolus*, vs. two robust setae in both sexes in the new species.

Two species of *Psammogammarus* are known only from a single female, but their morphology enables an easy separation from the new taxon. Thus, *Psammogammarus scopulorum* Stock, 1983, a coarse sand bar inhabitant of Los Roques archipelago in Venezuela differs markedly from the new species in the telson armature, devoid of lateral robust setae; the reduced marginal armature on both protopod and rami of U1 and U2; the comparatively longer U3 endopod (attaining 96% length of proximal segment of exopod, vs. 57% in the new species); the relative length of the U3 exopodal segments, with exp1 longer than exp2 (vs. the reverse in the new species); and the armature of epimeral plates, with 0-1-1 robust setae compared to 1-(2 or 3-3) in the new species (see Table 1; Stock 1983).

*Psammogammarus bluefieldensis* Ortiz, Lalana & Beltrán, 1993, from shallow muddy bottoms of the Caribbean coast of Nicaragua, differs from the rest of members of the genus in the display of a comparatively shallowly excavated telson (cleft only to almost midway compared to almost at base in the rest of species) and a 3-articulate accessory flagellum of antennule. In addition, it differs from the new species in the U1 and U2 rami, devoid of marginal armature; the shorter U3 endopod (attaining only 34% length of exp1, vs. 57% in the new species); and the U3 exp2, much shorter than exp1 (63% length of exp1, vs. exp2 longer than exp1 in the new species), among other distinctive features (see Table 1).

The new species from Indonesia displays a faint sexual dimorphism on pleopod II, where the male displays a rake conformed of short and blunt curved spinules along the medial margin of the proximal article of the exopod (Fig. 7B, C). Vonk (1990: 274 and fig. 1f) also noticed the presence of a unusual swelling placed in exactly the same position in the male pleopod II of *Psammogammarus stocki* Vonk, 1990. Furthermore, Stock and Vonk (1987: 246) stated “(male) pleopods normally segmented, not transformed”in the description of *Psammogammarus longidactylus*, and the same holds for *Psammogammarus caesicolus* Stock, 1980 (Stock 1980: 377). We have checked directly for the condition of pleopod II in at least two species of *Psammogammarus* of which we had material available for study. *Psammogammarus burri* Jaume & Garcia, 1993, a member of the cluster of species characterised by the display of a male G2 with an evenly convex, non-excavated palm margin and an elongated carpus, displays a non-sexually dimorphic pleopod, as stated in the original description. Likewise, a single male specimen of *Psammogammarus* cf. *caecus* –belonging to the cluster that displays a male G2 with a strongly excavated palm and a short, broader-than-long carpus– showed an unmodified pleopod II. This specimen was gathered with the other two at the Balearic Sea slope (552–1263 m depth) and represents the first record of the genus in deep waters (Cartes and Sorbe 1999). We thus discard that sexual dimorphism in pleopod II could be relevant in the taxonomic refinement of the genus, its value remaining limited to a mere species-level autapomorphic trait.

**Table 1.** Main diagnostic features of *Psammogammarus* species.

		*Psammogammarus wallacei* sp. n.	*Psammogammarus bluefieldensis*	*Psammogammarus burri*	*Psammogammarus coecus*	*Psammogammarus caesicolus*	*Psammogammarus garthi*	*Psammogammarus gracilis*	*Psammogammarus initialis*	*Psammogammarus longidactylus*	*Psammogammarus longiramus*	*Psammogammarus mawatarii*	*Psammogammarus scopulorum*	*Psammogammarus spinosus*	*Psammogammarus stocki*
1	male G2, outline of palm margin	excavated	?	convex	excavated	excavated	excavated	excavated	excavated	convex	convex	excavated	?	excavated	excavated
2	male G2, mid-palmar strong robust setae	0	?	3	1?	0	0	0	0	0	0	0	?	0	0
3	male G2, carpus	broader>long	?	longer>broad	broader>long	broader>long	broader>long	broader>long	broader>long	longer>broad	longer>broad	broader>long	?	broader>long	broader>long
4	U3 exopod, relative length of segments	exp2 > exp1	exp2 < exp 1	exp2 < exp1	exp2 > exp1	exp2 < exp1	exp2 = exp1	exp2 = exp1	exp2 > exp1	exp2 < exp1	exp2 < exp1	exp2 > exp1	exp2 < exp1	exp2 < exp1	exp2 = exp1
5	U3 end, % length of exp1	57	34	23	54	72	20	19	89	117	110	27	96	36	19
6	U1 protopod, basofacial robust setae	1	2	1	2	3	2	0	1	1	1	1	1	1	0
7	U2 protopod, robust setae on distolateral angle	1	1	1	1	3	1	0	1	0	1	1	1	1	1
8	U2 protopod, robust setae on distomed. angle	4	2	3	2	4	2?	1	4	2	2	2	2	2	1
9	telson, lateral robust setae	2	0	3	2 or 3	3	1–2 setae	1	3	2	4	1	2	2	3
10	telson, distal robust setae	1	?	0	2 or 3	1	2 setae	2	3–4 spines	2	2	1	0	2	0
11	epimeral plate III, posteroventral angle	weakly produced	strongly produced	quadrate	strongly produced	quadrate	strongly produced	rounded	weakly produced	weakly produced	weakly produced	rounded	strongly produced	rounded	rounded
12	epimeral plates, ventral margin (robust setae)	1-(2 or 3)-3	?	2-4-4	0-0-0	0-1-1	?	0-0-0	0-1-2	0-1-2	?	0-0-0	0-1-1	0-0-0	1-1-1
13	maxillule, setae on coxal endite	6	4	19	7	6	5	5	14	14	15	5	7	5	4
14	maxillule, basal endite (robust setae)	9	7	16	9	9	9	7	9	9	9	9	9	7	7
15	maxilla inner lobe, no. setae on oblique row	4	3	16	6	5	5	3	15	12	12	4	5	4	4
16	maxilliped, basal endite (robust setae)	3	3	3	3	6	3	0	3	3	3	3	3	3	0
17	maxilliped, ischial endite (robust setae)	4	?	7	7	4	0?	?	11	6	8	4	4	4	0
18	P7 basis, outline	weakly expanded	weakly expanded	weakly expanded	weakly expanded	weakly expanded	weakly expanded	weakly expanded	moderately expanded	broadly expanded	moderately expanded	weakly expanded	weakly expanded	weakly expanded	weakly expanded
19	P7 basis, margins	subparallel	subparallel	subparallel	subparallel	subparallel	subparallel	subparallel	convex	convex	subparallel	subparallel	subparallel	subparallel	subparallel
20	pleopod II, sexual dimorphism	yes	?	No	No	no	?	?	no	no	?	?	?	?	yes?

## Discussion

Ternate is situated in the centre of maximum marine species diversity, the Coral Triangle (Hoeksema 2007). The ranges of many marine benthic species overlap in this area. Most of these species have a larval phase during which dispersal through currents takes place. The low sea level stand during the Last Glacial Maximum (LGM) and the direction of inter-oceanic currents from the Pacific to the Indian Ocean are considered important in determining the ranges of reef coral species (Hoeksema 2007).

Because subterranean amphipods are known to be poor dispersers, their distribution patterns are expected to depend more on plate tectonics than on oceanic currents (Holsinger 1991; Stock 1993; Myers and Lowry 2009). Nevertheless, the subterranean beach environments may be very dynamic, even at secluded spots, and their fauna may move with the sediment, suggesting at least some dispersal within coastal areas (Vonk and Sánchez 1993; Vonk and Nijman 2006). However, the present study on *Psammogammarus wallacei* sp. n. and its closest relatives suggests that the fauna of southeastern Asia is still very poorly known (cf. Holsinger 1993), despite recent studies (e.g. Myers and Lowry 2009).

## Supplementary Material

XML Treatment for Psammogammarus wallacei
